# Metformin ameliorates arsenic trioxide hepatotoxicity via inhibiting mitochondrial complex I

**DOI:** 10.1038/cddis.2017.482

**Published:** 2017-11-02

**Authors:** Sunbin Ling, Qiaonan Shan, Peng Liu, Tingting Feng, Xuanyu Zhang, Penghui Xiang, Kangchen Chen, Haiyang Xie, Penghong Song, Lin Zhou, Jimin Liu, Shusen Zheng, Xiao Xu

**Affiliations:** 1 Division of Hepatobiliary and Pancreatic Surgery, Department of Surgery, Collaborative Innovation Center for Diagnosis and Treatment of Infectious Diseases, The First Affiliated Hospital, Zhejiang University School of Medicine, Hangzhou, China; 2 Key Laboratory of Combined Multi-Organ Transplantation, Ministry of Public Health, Hangzhou, China; 3 Key Laboratory of Organ Transplantation, Hangzhou, Zhejiang Province, China; 4 Department of Cancer Biology, University of Pennsylvania Perelman School of Medicine, Philadelphia, PA, USA; 5 Department of Abdominal Medical Oncology, Zhejiang Cancer Hospital, Hangzhou, China; 6 Department of Pathology and Molecular Medicine, Faculty of Health Sciences, McMaster University, Hamilton, Ontario, Canada

## Abstract

Arsenic trioxide (ATO) is a well-accepted chemotherapy agent in managing promyelocytic leukemia. ATO often causes severe health hazards such as hepatotoxicity, dermatosis, neurotoxicity, nephrotoxicity and cardiotoxicity. The production of reactive oxygen species, (ROS) play a significant role in ATO-induced hepatotoxicity. The oral hypoglycemic drug, metformin, is considered to be a potential novel agent for chemoprevention in the treatment of cancer. Moreover, metformin has also been shown to have hepatoprotective effects. In the present study, we demonstrated that metformin protected normal hepatocytes from ATO-induced apoptotic cell death *in vitro* and *in vivo*. Gene expression screening revealed that glucose metabolism might be related to the metformin-induced protective effect on ATO-treated AML12 cells. The metformin-promoted or induced glycolysis was not responsible for the protection of AML12 cells from ATO-induced apoptotic cell death. Instead, metformin increased the intracellular NADH/NAD+ ratio by inhibiting mitochondrial respiratory chain complex I, further decreasing the intracellular ROS induced by ATO. Treatment with low glucose or rotenone, a mitochondrial respiratory chain complex I inhibitor, also protected AML12 cells from ATO-induced apoptotic cell death. We show for the first time that metformin protects the hepatocyte from ATO by regulating the mitochondrial function. With its properties of chemoprevention, chemosensitization and the amelioration of liver damage, metformin has great prospects for clinical application other than type 2 diabetes mellitus (T2DM).

Arsenic trioxide (As_2_O_3_, ATO) has long been used as a therapeutic agent for certain severe diseases including malaria, syphilis, leukemia, tuberculosis and so on in East Asia, especially in China.^1^ According to recent guidelines in treating newly diagnosed or relapsed promyelocytic leukemia (APL), ATO alone or in combination with other therapeutic agents plays a significant role in remission induction and consolidation.^2, 3^ Furthermore, ATO can also be used to treat other hematological malignancies and various solid malignant tumors since ATO is well-known to induce apoptosis and result in cell cycle arrest.^4^ However, ATO often causes severe side effects, that is, hepatotoxicity, dermatosis, neurotoxicity, nephrotoxicity and cardiotoxicity,^5^ limiting its clinical application. Liver is one of the major target organs of ATO,^6^ whose exposure could produce reactive oxygen species (ROS), which could lead to oxidative damage to the liver tissue.^7^ Two important recent clinical trails in APL treatment reported that grade 3–4 liver toxicity was found in 25–63% of patients treated with ATO and all trans retinoic acid.^8, 9^

Metformin, a first line oral hypoglycemic drug for type 2 diabetes mellitus (T2DM), is considered to be a potential novel agent for chemoprevention in the treatment of cancer.^10, 11^ Accumulating evidence displayed that metformin could enhance cancer cells’ sensitivity to radiation and chemotherapy,^11, 12, 13^ such as the target drug, EGFR-TKI, in the treatment of patients with advanced non-small cell lung cancer.^14^ On the other hand, metformin is expected to have protective effects on oxidative stress.^15, 16^ Since metformin has been confirmed to have a cardiovascular protective effect in T2DM,^17, 18^ the potential role of metformin in protecting normal tissues and organs from oxidative stress and injury due to chemotherapy or radiation is also emerging.^19^ Metformin could significantly improve the survival of liver cirrhosis patients with diabetes.^20^ Moreover, metformin ameliorates acetaminophen hepatotoxicity in mice via reducing the ROS production.^21^ Several mechanisms responsible for metformin reducing oxidative stress have been described. Metformin could ameliorate high glucose-induced oxidative stress by inhibiting PKC-NAD(P)H oxidase pathway in human aortic endothelial cells.^22^ In elegans, metformin-induced ROS could promote the expression of peroxiredoxin PRDX-2 and consequently suppressed oxidative stress.^23^

Our group has previously reported that the combination of metformin and ATO has a synergistic anti-proliferative effect on certain hepatobiliary malignancies.^24, 25^ In normal liver, we hypothesized that metformin could decrease ATO-induced ROS production to reduce hepatotoxicity, which might lead to a new strategy of adding metformin to patients receiving ATO treatment in certain clinical settings.

However, understanding the mechanisms by which metformin protects the liver from injury is largely limited. There are significant differences in cell metabolism between normal cells and malignant cells,^26, 27^ and metformin is an agent regulating cellular glucose metabolism. In the present study, we evaluated the protective effects of metformin on ATO-induced hepatotoxicity *in vitro* and *in vivo*, and explored the altered glucose metabolism induced by metformin in mouse liver cells to demonstrate the underlying protective mechanisms of metformin on ATO-induced hepatotoxicity.

## Results

### Metformin protected AML12 cells from ATO-induced apoptotic cell death

**Figure 1:**
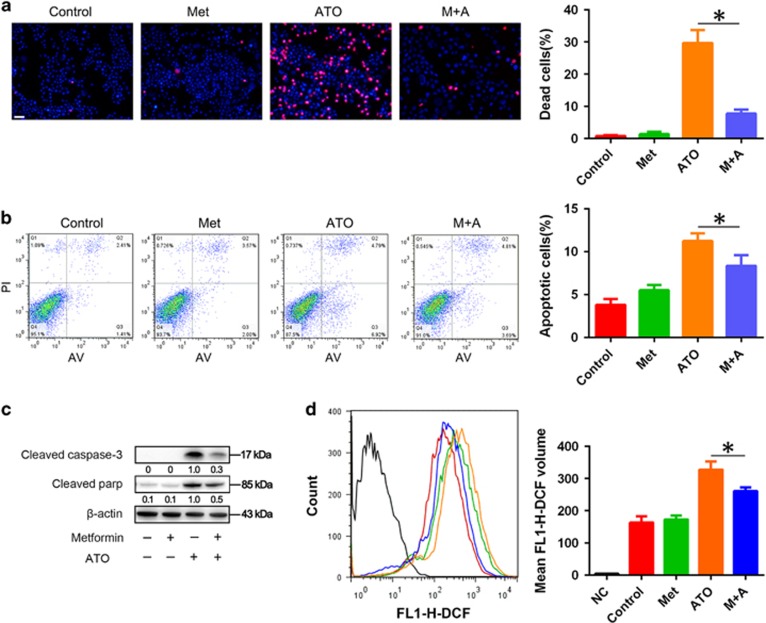
Protective effects of metformin on ATO-induced apoptotic cell death in AML12 cells. After treatment with 5 mM metformin and 6 *μ*M ATO in combination or single treatments for 48 h, (**a**) AML12 cells were stained by Hoechst 33342/PI and observed by fluorescence microscopy (× 200). AML12 cells were treated with 5 mM metformin and 6 *μ*M ATO in combination or single treatments for 24 h. (**b**) Then, the cells were examined using Annexin V/PI staining, and the distribution of apoptotic cells was measured by flow cytometry analysis. (**c**) Cleaved caspase-3 and cleaved PARP were monitored using western blot analysis. Band intensities were semi-quantified using Image Lab 5.0 software and normalized with *β*-actin. Values are represented as the means under the bands. (**d**) The intracellular ROS was measured by flow cytometry analysis using an oxidation‐sensitive fluorescent probe, DCFH-DA, which can be oxidized to DCF by ROS (the negative control was not treated with DCFH-DA). The experiments were repeated 3 times independently, and the bars represent the means±S.D. (*P<0.05). Scale bar, 100 *μ*m

The effect of metformin on the ATO-induced AML12 cell death was evaluated by Hoechst 33342/PI staining, for assessing cell apoptosis and intracellular ROS. ATO (6 *μ*M) could induce about 10% apoptosis in 24 h treatment and about 30% cell death in 48 h treatment in AML12 cells. Metformin could markedly reduce ATO-induced cell death and apoptosis in AML12 cells (Figure 1a–c). The intracellular ROS evaluation revealed similar results in AML12 cells treated with metformin and/or ATO (Figure 1d). The results implied that metformin could reduce ATO-induced cell death through the inhibition of apoptosis and decrease intracellular ROS in AML12 cells.

### Metformin alleviated ATO-induced liver injury

**Figure 2:**
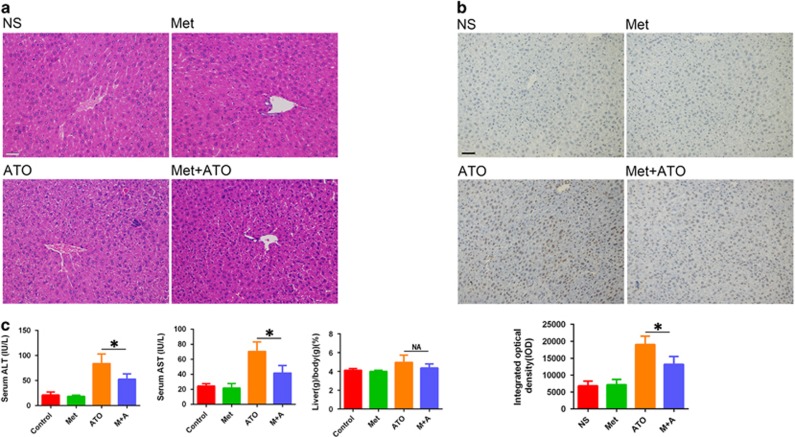
Effects of metformin on ATO-induced liver injury in kuming mice. (**a**) HE staining (× 200) of livers obtained from mice of different groups. (**b**) Apoptotic cells in the livers of mice were detected by the TUNEL assay. The data were quantified and are represented as the means±S.D. (**c**) The ALT and AST in the serum from mice of different groups were detected, and the values are presented are the means±S.D. (*n*=6 in each group; *P<0.05). Scale bar, 100 *μ*m

We used a mouse model to evaluate the effect of metformin on ATO-induced liver injury *in vivo*. We first observed obvious histopathologic changes in livers from ATO-treated mice compared to the control and metformin-treated group. In ATO-treated mice, liver revealed swollen hepatocytes with occasional apoptosis, which were attenuated by added metformin (Figure 2a). Moreover, TUNEL staining confirmed metformin could reduce ATO-induced cell apoptosis *in vivo* (Figure 2b). We further tested serum ALT and AST levels. As shown in Figure 2c, comparing to ATO, metformin+ATO effectively reduced serum ALT (from 83.7±18.3 IU/l to 52.3±10.2 IU/l, *P*<0.05) and AST (from 70.3±12.7 IU/l to 41.7±10.0 IU/l, *P*<0.05) levels, which concurred with the histopathologic findings. Although not statistically significant, the liver to body weight ratio tended to be higher in the ATO group compared to in other groups (ATO *versus* combination, 4.96±0.77% compared to 4.36±0.42%, *P*=0.132), which implied that ATO could induce hepatomegaly in mice, and metformin could effectively alleviate these effects. Collectively, the *in vivo* results confirmed the role of metformin in protecting ATO-induced liver cell apoptotic death.

### Gene expression screening revealed that glucose metabolism might be behind the metformin-induced protective effect on ATO-treated AML12 cells

**Figure 3:**
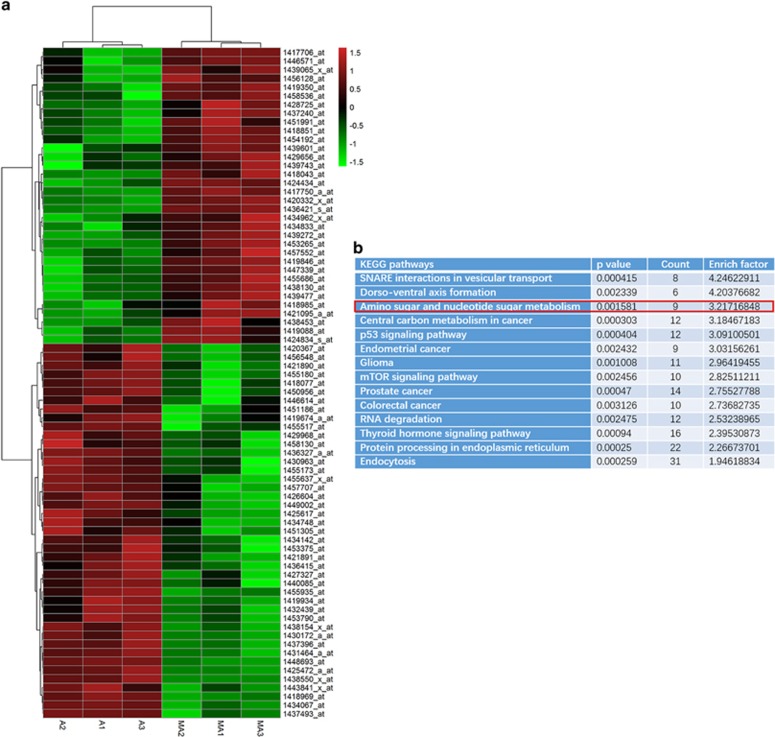
Effects of the different regulated genes and pathways of metformin on the ATO-treated AML12 cells. AML12 cells treated with ATO (6 *μ*M) and ATO (6 *μ*M)+metformin (5 mM) for 48 h were further applied for gene screening using mouse gene expression microarrays. (**a**) Changed genes in the AML12 cells of the two groups displayed by a heat map. (A 1–3=ATO group 1-3, MA 1-3=ATO+metformin group 1–3) (**b**) The different genes were analyzed and displayed by KEGG pathways. The pathway we were interested in is marked by the red pane

Aiming to explore the mechanism underlying the metformin-induced protective effect on ATO-treated AML12 cells, we examined the gene expression changes using mouse gene expression microarrays (Affymetrix Mouse Genome 430 2.0). The results showed that 77 genes (ATO+metformin *versus* ATO, 34 upregulated and 43 downregulated genes, *P*<0.005, representing a twofold difference in expression levels, Figure 3a), closely related with vesicular transport and cell metabolism were identified (Figure 3b). As vesicular transport is strongly correlated with cell autophagy,^28^ we first detected the autophagy status in AML12 cells treated with metformin and/or ATO. However, increased autophagy was only found in cells of the ATO group (data not shown), which implied that the autophagy is only an endogenous resistant effect of cell death and is triggered by ATO. Metformin did not promote the autophagy status when combined with ATO in AML12 cells. Thus, given that metformin is an agent targeting cell glucose metabolism and the KEGG analysis displayed the differences in the cell metabolic pathway (Figure 3b), we focused on the glucose metabolism regulation of metformin and ATO on AML12 cells in the following studies.

### Metformin suppressed mitochondrial oxidative phosphorylation and promoted glycolysis in AML12 cells

**Figure 4:**
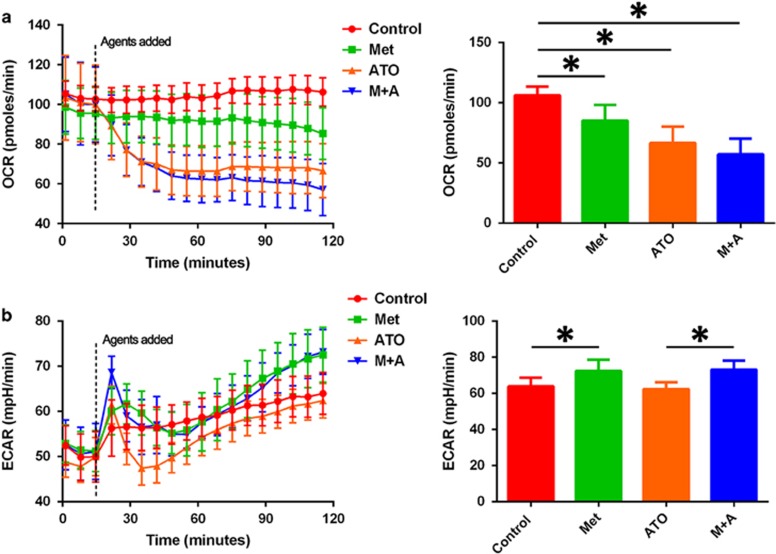
Effects of metformin and/or ATO treatment on changes in the glucose metabolism of AML12 cells. (**a**) The OCR and (**b**) ECAR in the four groups of AML12 cells were detected, and the values of the last detection are represented as the means±S.D. (6 replicates for per group; *P<0.05)

By using a Seahorse system, we further explored the effect of metformin and/or ATO on the glucose metabolism of AML12 cells. As displayed in Figures 4a and b, metformin significantly decreased the oxygen consumption rate (OCR) and increased the extracellular acidification rate (ECAR) in AML12 cells. The OCR reflects the mitochondrial oxidative phosphorylation (OXPHOS), and the ECAR is an indicator of glycolytic conversion of glucose to lactate.^29^ ATO significantly triggered OCR, which could be explained by the mitochondrial toxicity triggered by ATO-induced ROS,^30^ while ATO had no effect on ECAR in AML12 cells. Although no significant difference was found, a trend of decreased OCR was identified in the combination group in AML12 cells compared to the single ATO group (57.1±13.1 pmoles/min *versus* 66.6±13.5 pmoles/min, *P*=0.175).

### The promotion of glycolysis by metformin was not responsible for the protection of AML12 cells from ATO-induced apoptotic cell death

**Figure 5:**
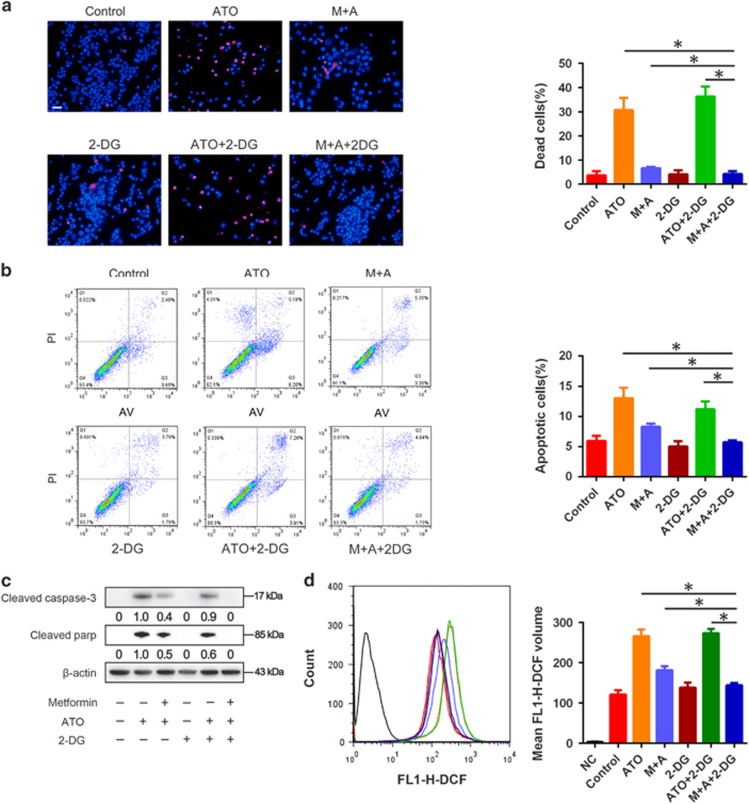
Influence of 2-DG on the protective effects of metformin on ato-induced apoptotic cell death in AML12 cells. The glycolysis of AML12 cells treated with ATO and metformin+ATO was abrogated by 2-DG treatment. (**a**) AML12 cells were stained by Hoechst 33342/PI and observed by fluorescence microscopy (× 200) after 48 h of treatment. (**b**) AML12 cells were examined using Annexin V/PI staining, and the distribution of apoptotic cells was measured by flow cytometry analysis after 24 h of treatment. (**c**) Cleaved caspase-3 and cleaved PARP were monitored using western blot analysis. Band intensities were semi-quantified using Image Lab 5.0 software and normalized with *β*-actin. Values are represented as the means under the bands. (**d**) The intracellular ROS was measured by flow cytometry analysis using an oxidation‐sensitive fluorescent probe, DCFH-DA, which can be oxidized to DCF by ROS (the negative control was not treated with DCFH-DA). The experiments were repeated three times independently, and the bars represent the means±S.D. (*P<0.05). Scale bar, 100 *μ*m

Given that aberrantly increased glycolysis in cancer cells was reported to be crucial for cellular antioxidant responses^31^ and resistance to chemotherapy,^32^ we hypothesized that the increase of glycolysis induced by metformin was responsible for the inhibition of ATO-induced apoptotic death in AML12 cells. However, when combined with a specific glycolysis inhibitor 2-deoxy-D-glucose (2-DG), the apoptotic cell death was abrogated in the metformin and ATO combination group in AML12 cells (Figure 5a and c). The ROS results were consistent with the cell death and apoptosis results (Figure 5d),and this is abnormal and interesting. What we could conclude is that the metformin-promoted glycolysis is not responsible for the inhibition of ATO-induced apoptotic death in AML12 cells. The OCR and ECAR variation under 2-DG treatment in AML12 cells was detected by Seahorse and is shown in Supplementary Figure S1.

### Treatment with low glucose or rotenone, a mitochondrial respiratory chain complex I inhibitor, protected AML12 cells from ATO-induced apoptotic cell death

**Figure 6:**
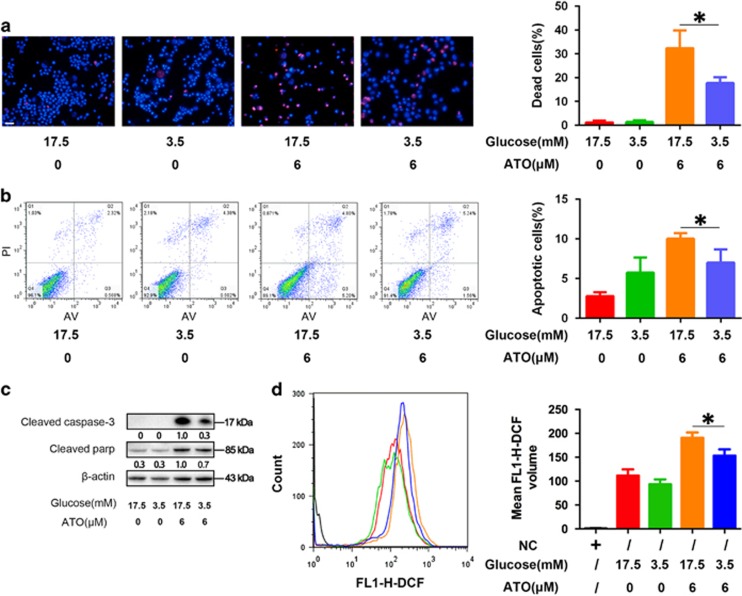
Protective effects of low glucose on ATO-induced apoptotic cell death in AML12 cells. After treatment with 6 *μ*M ATO with normal media (containing 17.5 mM glucose) or low glucose media (containing 3.5 mM glucose) for 48 h, (**a**) AML12 cells were stained by Hoechst 33342/PI and observed by fluorescence microscopy (× 200). AML12 cells were treated with 6 *μ*M ATO with normal media or low glucose media for 48 h. (**b**) Then, the cells were examined using Annexin V/PI staining, and the distribution of apoptotic cells was measured by flow cytometry analysis. (**c**) Cleaved caspase-3 and cleaved PARP were monitored using western blot analysis. Band intensities were semi-quantified using Image Lab 5.0 software and normalized with *β*-actin. Values are represented as the means under the bands. (**d**) The intracellular ROS was measured by flow cytometry analysis using an oxidation‐sensitive fluorescent probe, DCFH-DA, which can be oxidized to DCF by ROS (the negative control was not treated with DCFH-DA). The experiments were repeated three times independently, and the bars represent means±S.D. (*P<0.05). Scale bar, 100 *μ*m

**Figure 7:**
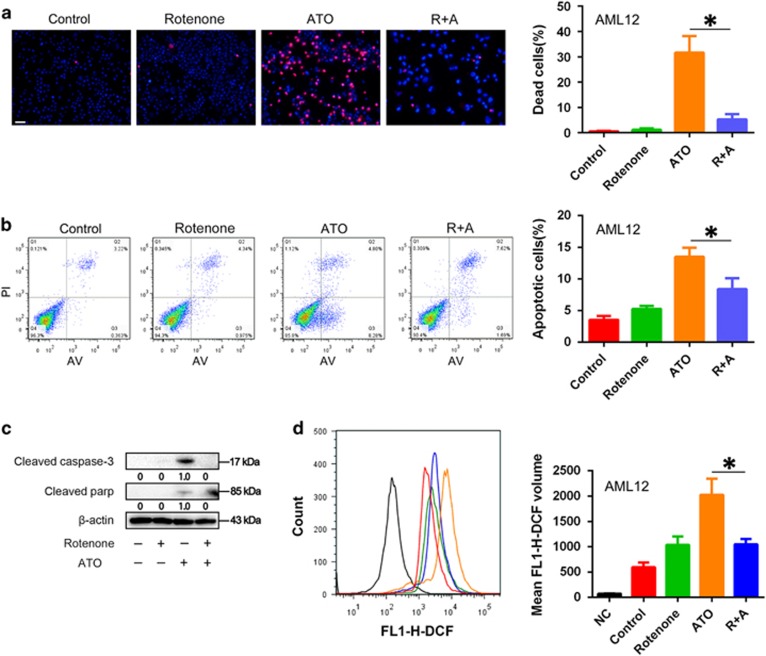
Protective effects of rotenone on ATO-induced apoptotic cell death in AML12 Cells. After treatment with 1 *μ*M rotenone and 6 *μ*M ATO in combination or single treatments for 48 h, (**a**) AML12 cells were stained by Hoechst 33342/PI and observed by fluorescence microscopy (× 200). AML12 cells were treated with 1 *μ*M rotenone and 6 *μ*M ATO in combination or single treatments for 24 h. (**b**) Then, the cells were examined using Annexin V/PI staining, and the distribution of apoptotic cells was measured by flow cytometry analysis. (**c**) Cleaved caspase-3 and cleaved PARP were monitored using western blot analysis. Band intensities were semi-quantified using Image Lab 5.0 software and normalized with *β*-actin. Values are represented as the means under the bands. (**d**) The intracellular ROS was measured by flow cytometry analysis using an oxidation‐sensitive fluorescent probe, DCFH-DA, which can be oxidized to DCF by ROS (the negative control was not treated with DCFH-DA). The experiments were repeated 3 times independently, and the bars represent means±S.D. (*P<0.05). Scale bar, 100 *μ*m

We further deduced that the metformin-induced inhibition of mitochondrial OXPHOS might mediate the protective effect on ATO-induced apoptotic cell death in AML12 cells. By using the same *in vitro* assays, the treatments of low glucose (Figure 6a–d) or rotenone (Figure 7a–d), a mitochondrial respiratory chain complex I inhibitor, displayed similar protective effects on ATO-induced apoptotic cell death as those of metformin in AML12 cells. These results implied that metformin might protect AML12 cells from ATO-induced apoptotic cell death via the inhibition of mitochondrial respiration.

### Metformin-induced inhibition of mitochondrial respiratory chain complex I mediated a protective effect on ATO-induced apoptotic cell death in AML12 cells

**Figure 8:**
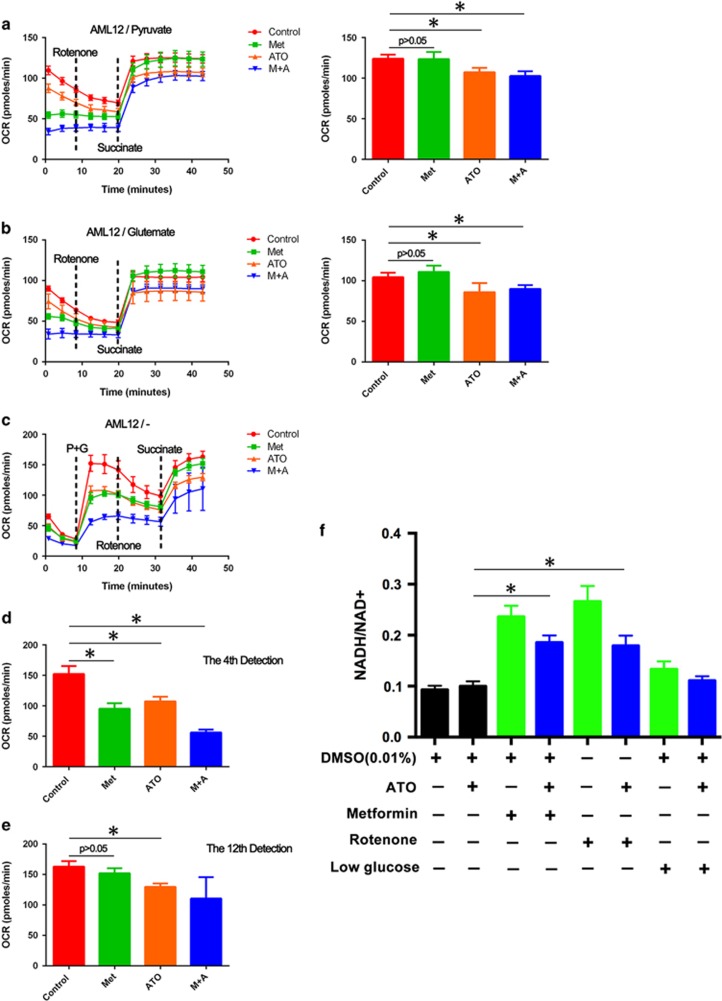
Effects of metformin and ATO on mitochondrial complex I and the NADH/NAD^+^ ratio in AML12 cells. The AML12 cells were pretreated with metformin (5 mM) and/or ATO (6 *μ*M) for 5 h. Then, the media was replaced by the modified DMEM (Seahorse Bioscience) with pyruvate (**a**) or glutamate (**b**). Rotenone was added to abrogate the function of complex I, and succinate was further added to activate the OCR. The values of the last detection are represented as the means±S.D. (6 replicates for per group). (**c**) The media was replaced by the modified DMEM (Seahorse Bioscience) without any substrate of the mitochondrial respiratory chain. Then, pyruvate and glutamate were added to activate the OCR of AML12 cells. Rotenone was added to abrogate the function of complex I, and succinate was further added to activate the OCR. The values of the fourth (**d**) and last (**e**) detections are represented as the means±S.D. (six replicates for per group). (**f**) The NADH/NAD^+^ ratios in AML12 cells treated with the agents ATO, metformin, rotenone, ATO+metformin and ATO+rotenone or cultured with low glucose DMEM/F12 and/or ATO for 24 h were detected. The values are represented as the means±S.D. (three replicates for per group; *P<0.05)

Given that metformin targeted mitochondrial complex I to perform its biological function,^33, 34^ the Seahorse system was used to evaluate the effect of metformin on mitochondrial respiratory chain complex I in AML12 cells. As shown in Figure 8a and b, cells were pretreated with metformin and/or ATO for 5 h, and by only supplying complex I substrate, the pyruvate or the glutamate in the media, the mitochondrial OXPHOS could only be initialed from complex I. The media was further added with rotenone to abrogate the function of complex I. At last, a complex II substrate, succinate, was added to activate the mitochondrial OXPHOS from complex II. Compared to the control group, no significant difference of OCR was found in the metformin group, but significant decreases were observed in the ATO and combination groups (decreases of 13.4% to 17.5% and 13.7% to 17.6%, respectively) in AML12 cells.

Moreover, as shown in Figure 8c, the OCR of AML12 cells pretreated with agent was abrogated initially in media without any substrate of the mitochondrial respiratory chain. After adding pyruvate and glutamate, a sharp increase of OCR was observed, but low levels were apparently found in the metformin, ATO and combination groups compared to the control group (Figure 8d). The results confirmed that both metformin and ATO targeted mitochondrial OXPHOS. Then, rotenone and succinate were further added to rescue the mitochondrial OXPHOS in AML12. Consistently, the OCR in the metformin group cells was revived to the level of the control group, while the OCRs in the ATO and combination groups were not (Figure 8e).

The OCR reduced by metformin could rescued by substrates of complex II but not the OCR reduced by ATO. Thus, these results revealed that metformin specifically targeted complex I which might mediated the protective effect on ATO-induced apoptotic cell death in AML12 cells. ATO might affect more mitochondrial complexes, as the OCR reduced by ATO could not be rescued by substrates of complex II, which could also explain the mitochondrial toxicity of ATO.^30^

### Metformin increased the intracellular NADH/NAD^+^ ratio of AML12 cells

**Figure 9:**
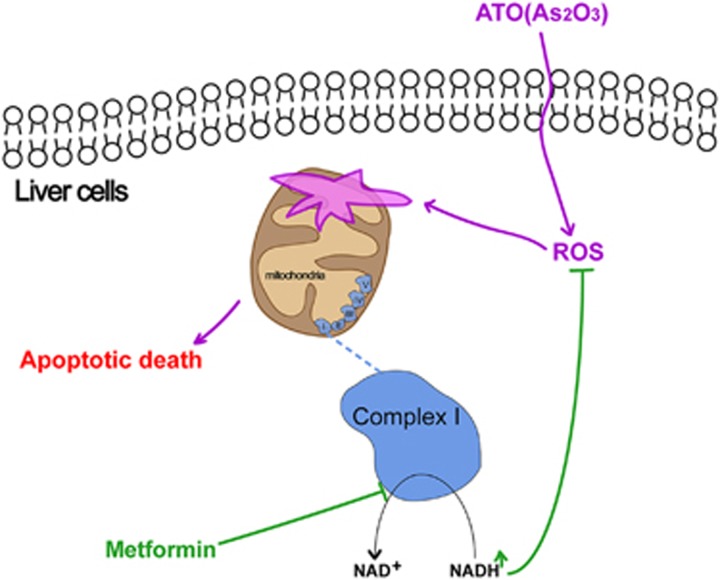
The possible mechanism of the mitochondrial complex I-dependent protective effect of metformin on ATO-induced hepatotoxicity. Metformin increased the intracellular NADH/NAD^+^ ratio by inhibiting mitochondrial respiratory chain complex I, further decreasing the intracellular ROS induced by ATO

When the mitochondrial respiratory chain complex I is working, electrons from NADH are passed to complex I, and NAD^+^ is formed. The NADH/NAD^+^ ratio will increase if the function of complex I is suppressed.^35^ Given that NADH plays a significant role in regulating intracellular ROS,^35^ we detected the NADH/NAD^+^ ratios in AML12 cells treated with the agents ATO, metformin, rotenone, ATO+metformin and ATO+rotenone or cultured with low glucose DMEM/F12 and/or ATO (Figure 8f). Ideally, both metformin and rotenone effectively increased the NADH/NAD^+^ ratio in AML12 cells treated with ATO. Therefore, metformin might increase the intracellular NADH/NAD^+^ ratio by targeting mitochondrial respiratory chain complex I, further decreasing the intracellular ROS induced by ATO and protecting AML12 cells from ATO-induced apoptotic cell death (Figure 9). However, only a slight increase of the NADH/NAD^+^ ratio in ATO-treated AML12 cells with low glucose media was observed, which might be explained by that low glucose is not specifically affecting complex I but instead the whole mitochondrial respiratory chain, the components of which, such as complex III, might affect the intracellular ROS and mediate the protective effect.^36^

## Discussion

ATO is a traditional Chinese medicine that is mainly used to treat acute APL. In a recent phase 3 trail for APL treatment, grade 3–4 liver toxicity was reported in 25% of patients in the ATRA and ATO group versus 10% of patients in the ATRA and idarubicin group.^9^ Moreover, ATO in combination with other chemotherapy or radiation might have clinical values in some solid malignant tumors.^37^ Recent studies were mainly focused on the chemoprevention and anti-tumor effect of metformin,^38^ including the studies we conducted, which revealed that metformin potentiated the effect of ATO in hepatobiliary malignancy.^24, 25^ As an inexpensive and widely accessible antidiabetic medication without severe adverse effects, metformin was reported as having a protective effect on normal tissue injured by chemotherapy, inflammation or other agents.^20, 21, 39^ Therefore, it is extremely valuable to elucidate the effects of the underlying mechanisms of metformin on normal tissues that are injured by ATO toxicity.

In this study, we used a concentration of ATO (6 *μ*M) to construct an *in vitro* liver cell injury model. A relative high concentration of ATO in anti-blood tumor researches is 6 *μ*M,^40, 41^ but commonly used in solid tumor researches.^42, 43, 44^ By *in vitro* and *in vivo* assays, we demonstrated the protective effects of metformin on ATO-induced liver injury. Metformin decreased ATO-induced ROS and apoptotic cell death in AML12 cells, which were commonly used in hepatotoxicity research.^45, 46^ The results of gene expression microarrays revealed that the alteration of glucose metabolism induced by metformin might mediate the protective effects.

The production of ROS plays a significant role in ATO-induced hepatotoxicity.^47, 48^ The accumulation of ROS mediated by ATO can induce the failure of the mitochondrial transmembrane potential, subsequently generating large amounts of ROS,^49, 50^ releasing cytochrome C and resulting in cell apoptosis.^30^ That partly explains why the organs or tissues with a larger mitochondrial content are more vulnerable to chemotherapy-induced injury. As is known, mitochondria are the key organelle participating in energy generation and modulating apoptosis. At the same time, mitochondria produce large amounts of ROS and are important participants in redox-dependent intracellular signaling.^51, 52^

There are five electron transport carriers, complexes I to V, embedded in the lipid bilayer of the inner mitochondrial membrane. Complex I (NADH-ubiquinone oxidoreductase) transfers electrons from NADH to ubiquinone (coenzyme Q, CoQ). The energy released by this process results in protons being transported across the mitochondrial membrane. In the presence of oxygen, electrons escaped from the mitochondrial electron transport chain could generate the superoxide anions, which are rapidly converted into hydrogen peroxide. Complex I is a major carrier of electron and subsequent superoxide production.^53^ In the present study, we used rotenone simulating the same protective effect in AML12 cells as metformin, while the inhibition of glycolysis by 2-DG failed to abrogate this phenomenon. These results revealed that the inhibition of mitochondrial respiratory chain complex I, but not the induction of glycolysis, mediated the protective effect of metformin on ATO-induced apoptotic cell death in mouse liver cells. Different with metformin or rotenone, ATO affected more mitochondrial complexes which is consistent the previous studies.^30^ Intriguingly, the combined use of metformin and 2-DG seemed to display a better protective effect compared with only metformin treatment, while 2-DG alone had no effect on ATO-induced apoptotic cell death in AML12 cells. These findings might imply other mechanisms involved in the protective effect. Moreover, an increased NAPH/NAD^+^ ratio was observed in AML12 cells treated with metformin or rotenone, which implies that metformin might increase the intracellular NADH/NAD^+^ ratio by inhibiting mitochondrial respiratory chain complex I. The above change could further decrease the intracellular ROS induced by ATO and protecting AML12 cells from ATO-induced apoptotic cell death. However, only a slight increase of the NAPH/NAD^+^ ratio was observed in AML12 cells with glucose-limited culture conditions. The low glucose concentration could inhibit both OXPHOS and glycolysis in AML12 cells (data not shown), which partly confirmed the protective effect of the combined treatment of metformin and 2-DG on ATO-induced liver cell apoptotic death. However, more experiments should be performed related to this issue.

Actually, some previous studies have reported that metformin could hamper ROS production induced by a reverse-electron flux at respiratory-chain complex 1^54^ and increased the NADH/NAD^+^ ratio.^55^ Our results confirm the previous findings and unfold the potential use of metformin in this special clinical setting. Given that some of the cancer stem cells were dependent on mitochondrial OXPHOS and could be specially targeted by metformin,^56, 57, 58^ metformin administered as an adjuvant agent might regulate the metabolism propensity of cancer stem cells and sensitize these cancer cells to chemotherapy.

The main shortcoming of this study is that the precise origin of ROS induced by ATO in AML12 cells is unclear. One of our previous studies demonstrated that metformin and ATO synergistically increased the intracellular ROS and apoptosis in cholangiocarcinoma cells,^25^ while an opposite effect was found in AML12 cells, a normal mouse liver cell line. Given the different metabolic backgrounds of cancer cells and non-cancerous cells,^26, 27^ our future studies will explore both the generation and regulation in the two types of cells treated with metformin and ATO.

In conclusion, this study revealed that metformin performed a protective effect for ATO-induced liver injury by targeting mitochondrial complex I and increasing the NADH/NAD^+^ ratio. Numerous chemotherapeutic agents including ATO could injure the normal tissues by inducing oxidative stress.^5, 7^ The liver, heart, kidneys and nervous system are easily damaged by chemotherapy. Our future studies will also explore the protective effect of metformin on other organs. Containing properties of chemoprevention, chemosensitization and the amelioration of tissue damage, metformin has great prospects in clinical application.

## Materials and methods

### Cell culture

Alpha mouse liver 12 (AML12) cells, a normal mouse liver cell line, was purchased from the Type Culture Collection of the Chinese Academy of Sciences (Shanghai, China) and cultured in DMEM/F12 (Gibco, Carlsbad, CA, USA) supplemented with 10% fetal bovine serum (FBS; Gibco), ITS liquid media supplement (Sigma, St. Louis, MO, USA), dexamethasone (40 ng/ml) and 100 *μ*g/ml each of penicillin and streptomycin (Invitrogen, Carlsbad, CA, USA) in 5% CO_2_ at 37 °C. The low glucose DMEM/F12 was a 1 : 1 mixture of no glucose DMEM (Gibco) and Ham's F-12 medium (Gibco).

### Reagents

Metformin (1,1-dimethylbiguanide hydrochloride, #D150959-5G) and rotenone (#R8875) were purchased from Sigma-Aldrich (St. Louis, MO, USA). Arsenic trioxide (As_2_O_3_, ATO) was purchased from the Shuanglu Pharmaceutical Co., Ltd. (Beijing, China). The Annexin V-FITC/PI Apoptosis Detection Kit (KGA108), the Hoechst 33342/PI Staining Kit (KGA212) and the ROS Detection Kit (KGT010) were purchased from KeyGen Biotech (Nanjing, China).

### Fluorescent cell survival assay

The agent toxicity was evaluated by a Hoechst 33342/PI staining assay.^59^ AML12 cells were seeded into 6-well plates at a density of 2 × 10^5^ cells/ml in the medium (2 ml) and incubated with metformin (5 mM) and/or ATO (6 *μ*M) for 48 h. Then, the cells were trypsinized and collected. After being stained with 2 *μ*g/ml Hoechst 33342 for 15 min at 37 °C, cells were washed by PBS and stained with 2 *μ*g/ml PI for 15 min at room temperature. The cell suspension was then loaded into the microslides and observed by fluorescence microscopy. The number of dead (PI positive) cells and the total number of PI positive+Hoechst 33342 positive cells were calculated.

### Cell apoptosis and ROS evaluation

The cell apoptosis and intracellular ROS levels were determined by flow cytometry. Portions of the detailed procedure have been described previously.^25^ All of the cells were detected after treatment with certain agents for 24 h.

### Western blot analysis and antibodies

Cells after different treatments were collected for western blot analysis. The detailed procedure has been described previously.^11, 60^ Primary antibodies were incubated at 4 °C overnight. The bands were visualized by chemiluminescence, imaged using a ChemiDoc XRS and analyzed using Image Lab (both from Bio-Rad). The following antibodies were used for immunoblotting: *β*-actin (sc-47778) was from Santa Cruz Biotechnology, Inc. (Santa Cruz, CA, USA); cleaved caspase-3 (#9661) was from Cell Signaling Technology, Inc. (Danvers, MA, USA); PARP1 (13371-1-AP) was from the Wuhan Sanying Company (Wuhan, China); and goat anti-rabbit and goat anti-mouse IgG peroxidase-conjugated secondary antibodies (31460 and 31430) were from Thermo-Pierce (Rockford, IL, USA).

### Mouse gene expression microarrays (Affymetrix Mouse Genome 430 2.0)

Mouse gene expression microarrays (Affymetrix U133 Plus 2 chip, Affymetrix, Santa Clara, CA, USA) were used to analyze the differential gene expression in response to 48 h of ATO or ATO+metformin treatment in AML12 cells. The experiment was mainly conducted by the Bohao Biotech Co., Ltd. (Shanghai, China). A heat map was performed based on Gene Ontology and KEGG to find differentially expressed gene pathways.

### Design of experiments using animals

Eight-week male Kunming mice weighing 35–40 g were obtained from the Animal Facility of Zhejiang University. The animals were maintained at 24±1 °C, 45±5% humidity and a 12 h light-12 h dark cycle. They were provided with standard laboratory chow and water *ad libitum*. The mice were randomly divided to four groups (*n*=6 each) as control (100 *μ*l of NS by intraperitoneal injection and 100 *μ*l of NS by gavage), metformin (200 mg/kg/day diluted in 100 *μ*l of NS by intraperitoneal injection and 100 *μ*l of NS by gavage), ATO (12 mg/kg/day diluted in 100 *μ*l of NS by gavage), and a combination of both agents (metformin, 200 mg/kg/day diluted in 100 *μ*l of NS by intraperitoneal injection plus ATO, 12 mg/kg/day diluted in 100 *μ*l of NS by gavage) groups. The treatment was conducted for consecutive 3 days.

Then, the mice were weighed and euthanized 24 h following the last administration of the agents. Blood samples were collected and centrifuged for 10 min at 3000 × *g*. The obtained clear serum was stored at −20 °C until alanine aminotransferase (ALT) and aspartate aminotransferase (AST) levels were measured using a Hitachi 7600 automatic analyzer (Hitachi, Tokyo, Japan). The liver was isolated and weighed. The use of mice in this study was approved by the Medical Ethics Committee of the First Affiliated Hospital of Zhejiang University.

### Histopathological examination

The liver obtained from each animal was fixed in a 10% formalin solution, processed according to routine protocol and embedded in paraffin block. Sections (10 *μ*m-thick) were taken and stained with hematoxylin and eosin (H&E). A pathologist unaware of the mice groups examined the slides under a light microscope.

### TUNEL assay

*In situ* detection of apoptotic cells in the livers isolated from the mice was performed with a TUNEL assay. The paraffin blocks of the livers were cut into 10 *μ*m-thick sections in a microtome cryostat. The TUNEL assay was conducted according to the manufacturer’s protocols. 3,3-Di-aminobenzidine (DAB) was used as the substrate for the peroxidase. Images were captured with a light microscope, and 5 images per sample were prepared. Image-Pro Plus 4.5 (Media Cybernetics, Silver Spring, MD, USA) Software was used to analyze the staining data.

### Monitoring of glucose metabolism by Seahorse

The glucose metabolism of AML12 cells was monitored by a Seahorse system. Cells seeded at 8 × 10^3^ cells per well were initially plated in XF assay medium, modified DMEM (Seahorse Bioscience Inc., Billerica, MA, USA) containing 25 mM glucose, 2 mM L-glutamine, and 1 mM sodium pyruvate, and incubated in a non-CO_2_ incubator at 37 °C for 30 min. OCR and ECAR were measured at 37 °C in an XF96 Extracellular Flux Analyzer (Seahorse Bioscience) using manufacturer-recommended protocols. After baseline measurements, the OCR and ECAR were measured sequentially.

For the detection of the effects of agents on mitochondrial respiratory chain complex I, a novel kit, XF PMP (Seahorse Bioscience, Cat # 102504-100), was used according to the protocol (http://www.agilent.com/cs/library/technicaloverviews/public/XF-PMP-Limited-Tech-Brief-WEB.pdf).

### NADH/NAD+ determination

Experiments to determine the NADH/NAD^+^ ratio in AML12 cells exposed to metformin and/or ATO were carried out using the NAD^+^/NADH assay kit from Abcam (ab65348, Cambridge, MA, USA) according to the manufacturer’s instructions. Briefly, AML12 cells were seeded into 6-well plates at a density of 2 × 10^5^ cells/ml in the medium (2 ml). After 24 h of pre-incubation, cells were divided into certain groups: control (0.01% DMSO in DMEM/F12), ATO (6 *μ*M ATO, 0.01% DMSO in DMEM/F12), metformin (5 mM metformin, 0.01% DMSO in DMEM/F12), metformin and ATO (5 mM metformin, 6 *μ*M ATO, 0.01% DMSO in DMEM/F12), rotenone (1 *μ*M rotenone in DMEM/F12), rotenone and ATO (1 *μ*M rotenone, 6 *μ*M ATO in DMEM/F12), low glucose (0.01% DMSO in low glucose DMEM/F12), and low glucose and ATO (6 *μ*M ATO in low glucose DMEM/F12). Exposure to each agent was allowed for 24 h. Then, cells were collected and washed by cold PBS. Cell pellets were extracted using the NAD/NADH extraction buffer and filtered using a 10-kDa-molecular weight cutoff filter (Abcam). Samples were split into two, with the first half being used to determine NADH and the second half used to determine the total NADt (NAD^+^+NADH). The absorbance at 450 nm was normalized against the protein content in each sample determined using the BCA assay (KeyGen Biotech, Nanjing, China). Three replicates were prepared for each condition, and the NADH/NAD^+^ ratio was calculated.

### Statistical analysis

SPSS 21.0 statistical software was used for the statistical analysis. Values are presented as the mean±S.D. Statistical analyses were performed using the Student’s *t*-test. The analysis of multiple groups was performed by ANOVA with an appropriate *post hoc* test.
