# Supplementary figures and images for: Metformin ameliorates arsenic trioxide hepatotoxicity via inhibiting mitochondrial complex I
Source: Cell Death Dis. 2017 Nov 2;8(11):e3159–. doi: 10.1038/cddis.2017.482 (PMC5775401; doi:10.1038/cddis.2017.482)

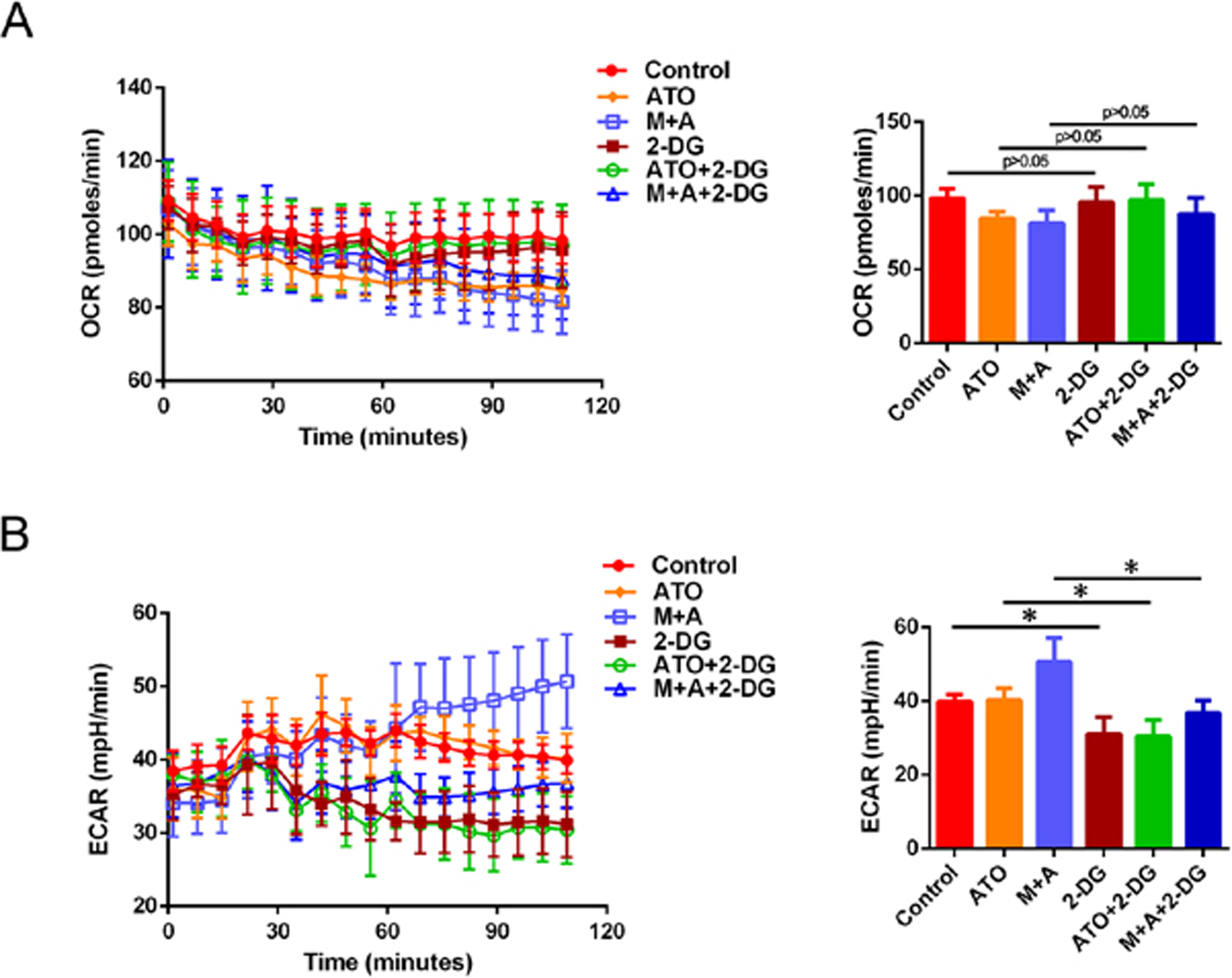

Supplement: Supplementary Figure S1 [file cddis2017482x1.tif]
