# Supplementary material for: Metformin ameliorates arsenic trioxide hepatotoxicity via inhibiting mitochondrial complex I
Source: Cell Death Dis. 2017 Nov 2;8(11):e3159–. doi: 10.1038/cddis.2017.482 (PMC5775401; doi:10.1038/cddis.2017.482)
Supplement: Supplementary Figure S1 Legend [file cddis2017482x2.doc]

**Supplemental information**

**Figure 1S: The OCR and ECAR variation under metformin, ATO and/or 2-DG treatment in AML12 cells.** (A) The OCR and (B) ECAR in the six groups of AML12 cells were detected, and the values of the last detection are represented as the means ± SD (6 replicates for per group, M+A = metformin + ATO) (^*^P < 0.05).
